# Supplementary material for: Exploring Alzheimer’s Disease Molecular Variability via Calculation of Personalized Transcriptional Signatures
Source: Biomolecules. 2020 Mar 26;10(4):503. doi: 10.3390/biom10040503 (PMC7226317; doi:10.3390/biom10040503)
Supplement: Supplementary file 1 [file biomolecules-10-00503-s001.zip › suppl/biomolecules-719963-supplementary-1.pdf]

Supplementary file

Exploring Alzheimer's Disease molecular variability via calculation of personalized transcriptional signatures

Hila Dagan[1#], Efrat Flashner-Abramson[2#], Swetha Vasudevan[2#], Maria R. Jubran[2], Ehud Cohen[3] and Nataly Kravchenko-Balasha[2]*

[1]The Rachel and Selim Benin School of Computer Science and Engineering, Hebrew University, Jerusalem, Israel

[2]Department for Bio-medical Research, Faculty of Dental Medicine, Hebrew University of Jerusalem, Jerusalem 91120, Israel

[3]Department of Biochemistry and Molecular Biology, The Institute for Medical Research Israel – Canada, The Hebrew University School of Medicine, Jerusalem, Israel

*Corresponding author: natalyk@ekmd.huji.ac.il
[#] Equal contribution

Figure S1

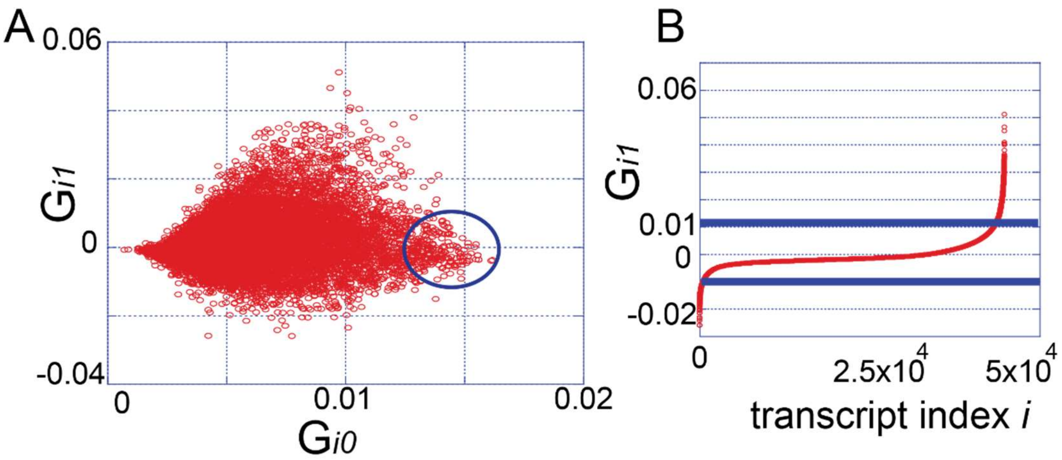

Fig. S1 Only transcripts with significant $G_{i\alpha}$ values are considered to participate in the steady state and unbalanced processes *α*. (A) G_{i0} values for all analyzed transcripts were plotted against corresponding G_{i1} values. The most stable transcripts, with $G_{i0} > 0.012$ (encircled in blue) have been found to participate less in the unbalanced processes as shown in **(A)**: stable transcripts have small weights $-0.01 < G_{i1} < 0.01$, meaning that they do not participate in the unbalanced process 1. The same result was obtained when G_{i0} values for all transcripts were plotted against corresponding $G_{i\alpha}$ values for all 7 processes (not shown). **(B)** The transcripts that take part in the different unbalanced processes were identified as follows: For every unbalanced process α, $G_{i\alpha}$ values were sorted according to their weight, and only transcripts with significant $G_{i\alpha}$ values were considered to participate in the unbalanced process α. This is exemplified for the process $\alpha = 1$ in the figure. Shown are sorted values of G_{i1}, which represent the degree of participation of every transcript *i* in the unbalanced process $\alpha = 1$. The blue lines represent threshold values. Transcripts with $G_{i1} > 0.01$ or $G_{i1} < -0.01$ (which are located on the top and bottom "tails" of the distribution) were considered to participate in the unbalanced process $\alpha = 1$. These transcripts were used to find a biological meaning of each unbalanced processes using David database, as presented in Supplementary Table 2.

Figure S2

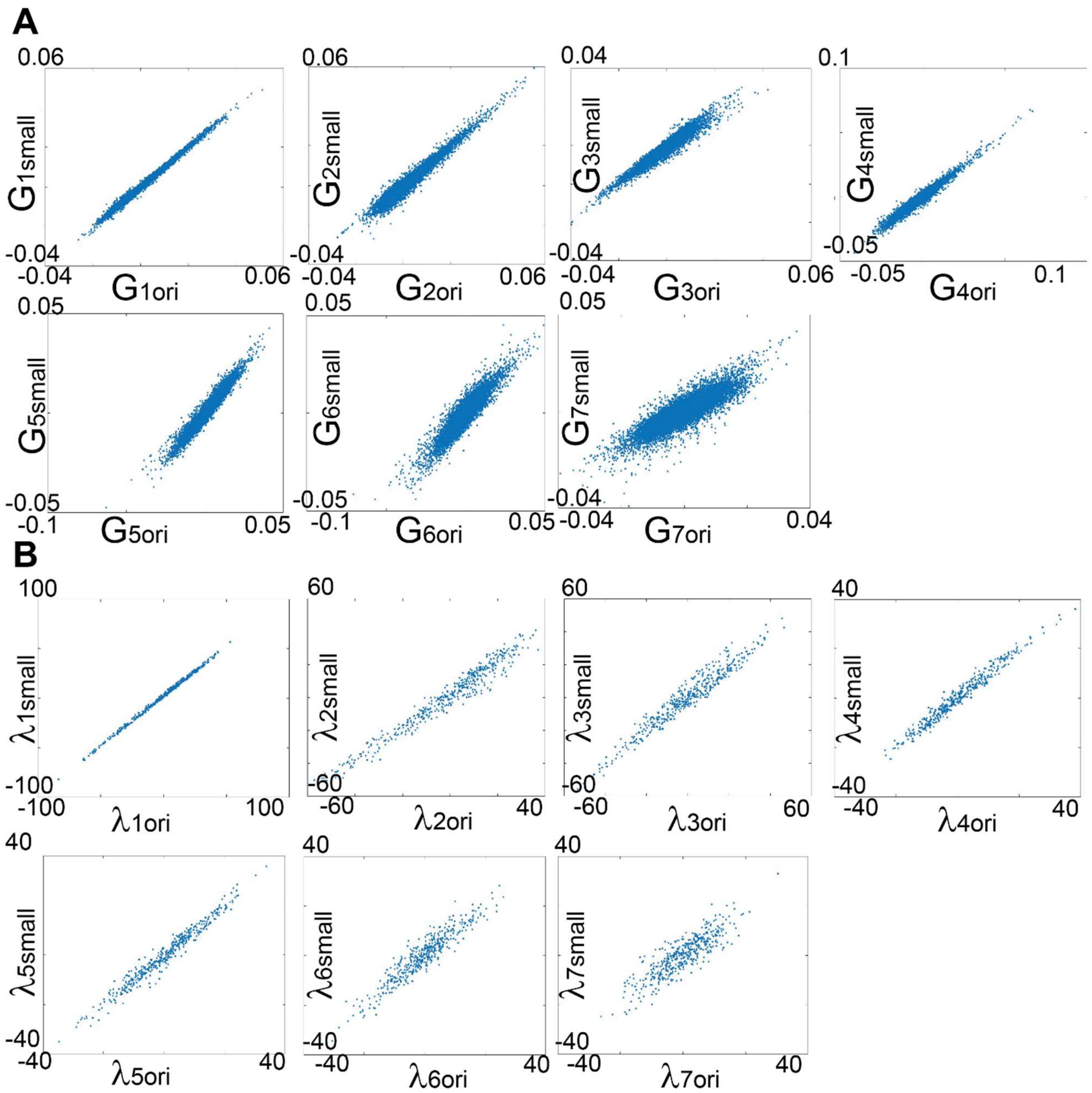

Fig. S2 Unbalanced processes remained the same when either the entire dataset or a subset of the dataset were used for the analysis. The weights of the transcripts (Giα; **A**) and the amplitudes of the unbalanced processes (λα; **B**) were found to be similar when either the entire dataset (**ori** dataset, 951 samples) or only half the population of patients (**small** dataset, 451 random samples) were analyzed, as indicated by high correlation of the scatter plots. The correlation coefficient R is at least 0.6 for all the processes.

Figure S3

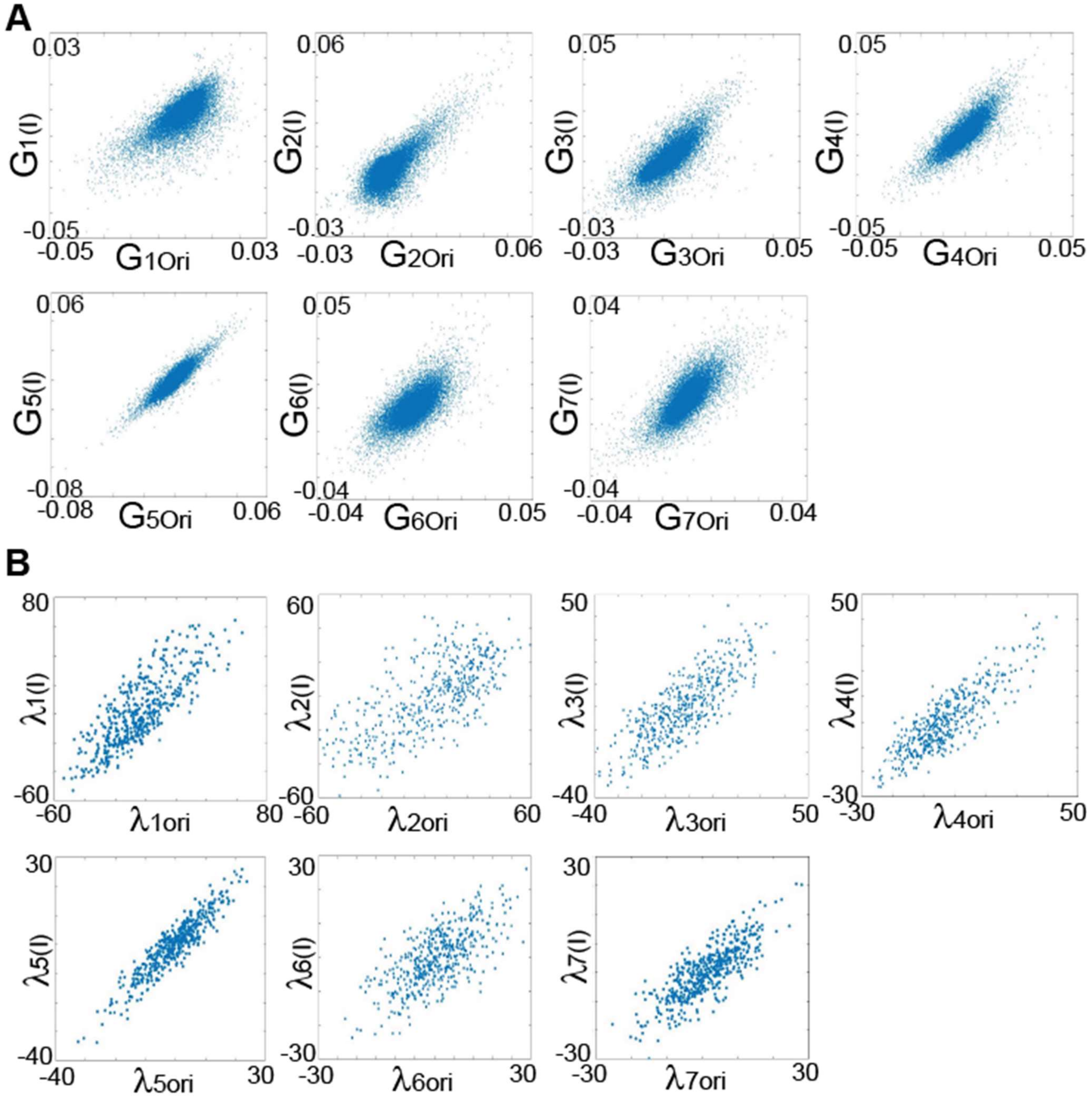

Fig. S3 Unbalanced processes remained the same when either the entire dataset or half of the dataset were used for the analysis. The original dataset was divided in two subsets. Each subset was analyzed separately and compared to original (**ori**) dataset. The weights of the transcripts (Giα; **A**) and the amplitudes of the unbalanced processes (λα; **B**) were found to be similar when either the entire dataset (**ori** dataset, 951 samples) or only half the population of patients (first subset (**I**) is shown here, 475 samples) were analyzed, as indicated by high correlation of the scatter plots. The correlation coefficient R is at least 0.6 for all the processes.

Figure S4

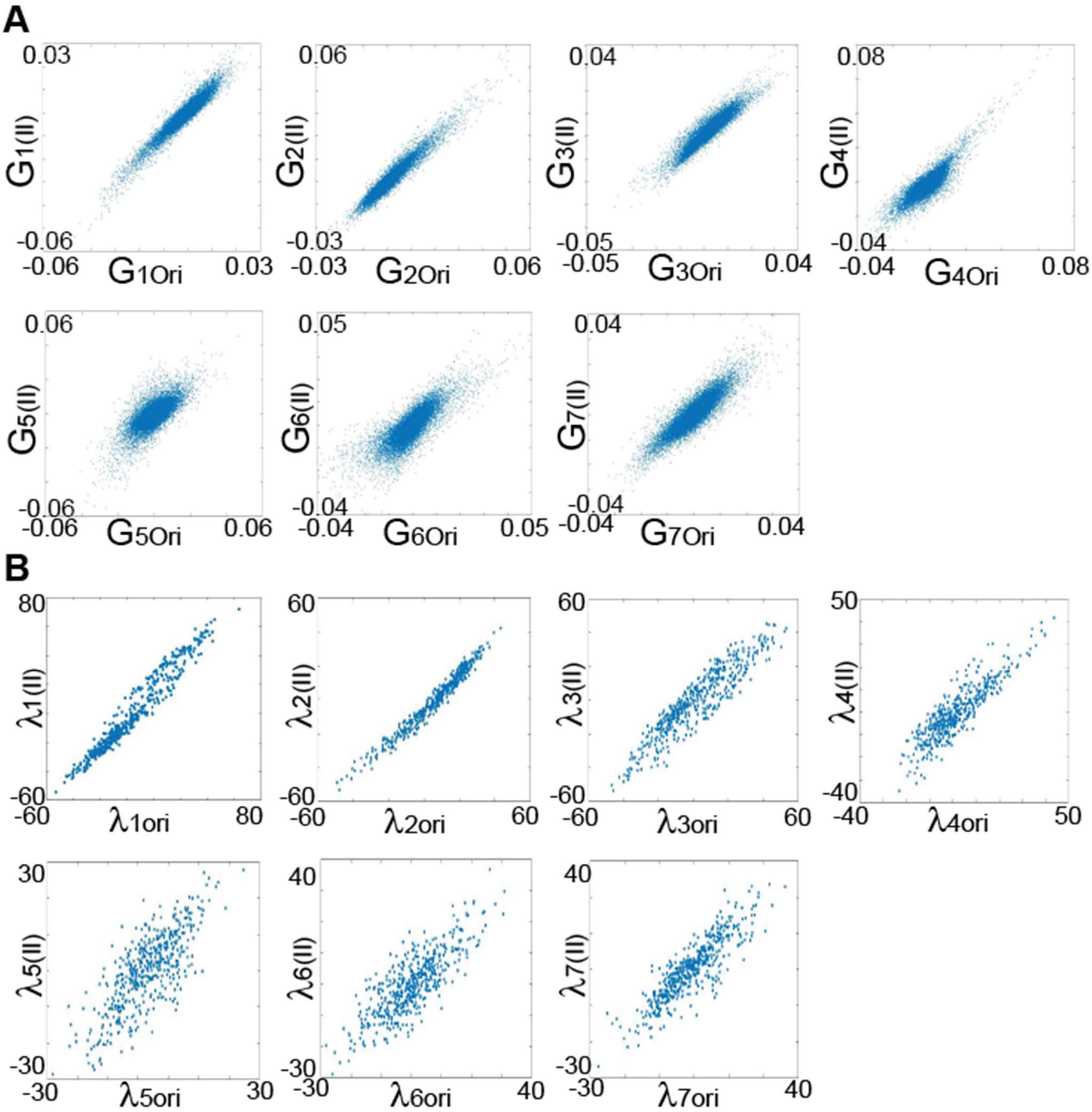

Fig. S4 Unbalanced processes remained the same when either the entire dataset or the second half of the dataset were used for the analysis. The original dataset was divided in two subsets. Each subset was analyzed separately and compared to original (**ori**) dataset. The weights of the transcripts ($Gi\alpha$; **A**) and the amplitudes of the unbalanced processes ($\lambda\alpha$; **B**) were found to be similar when either the entire dataset (**ori** dataset, 951 samples) or only half the population of patients (second subset (**II**) is shown here, 476 samples) were analyzed, as indicated by high correlation of the scatter plots. The correlation coefficient R is at least 0.6 for all the processes.

Figure S5

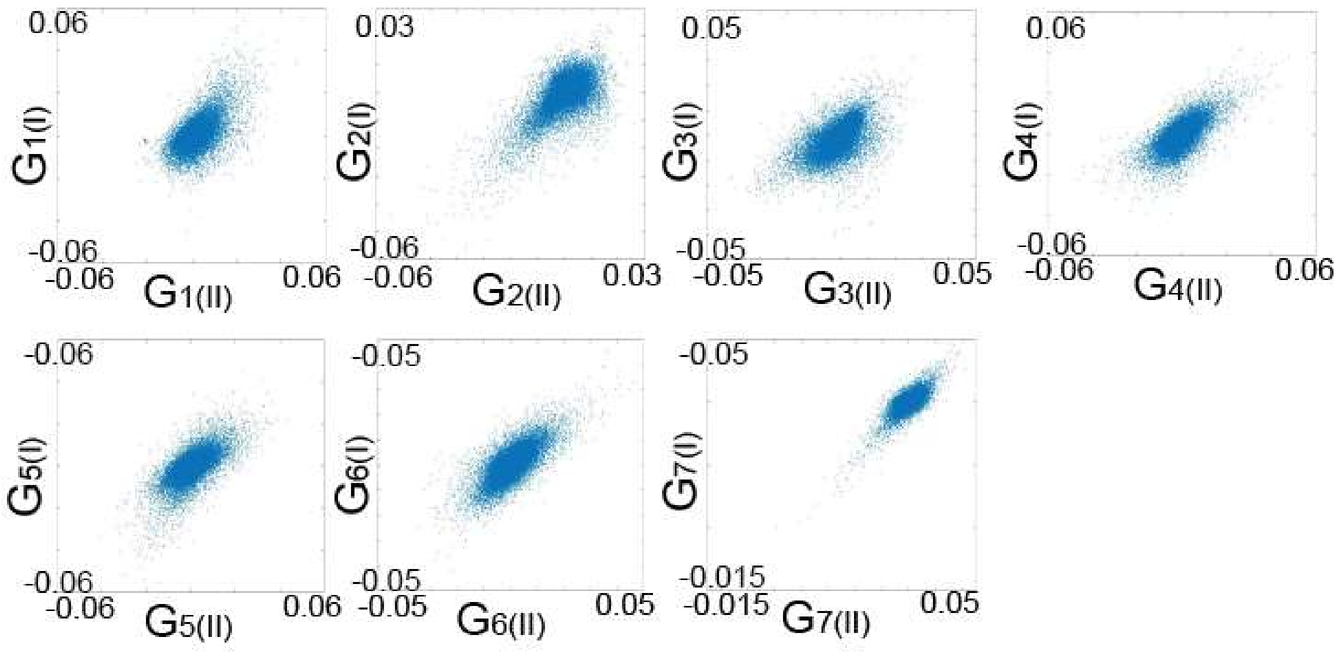

Fig. S5 Unbalanced processes remained the same when either the first half of the dataset (I) or the second half of the dataset (II) were analyzed separately. The original dataset was divided in two subsets. Each subset was analyzed separately, and the two subsets were compared using scatter plots of Giα values. The correlation coefficient R was at least 0.5 for all the processes. The weights of the transcripts (Giα) were found to be similar in each analysis, demonstrating that the same unbalanced processes were identified when the subsets were analyzed independently.

Figure S6

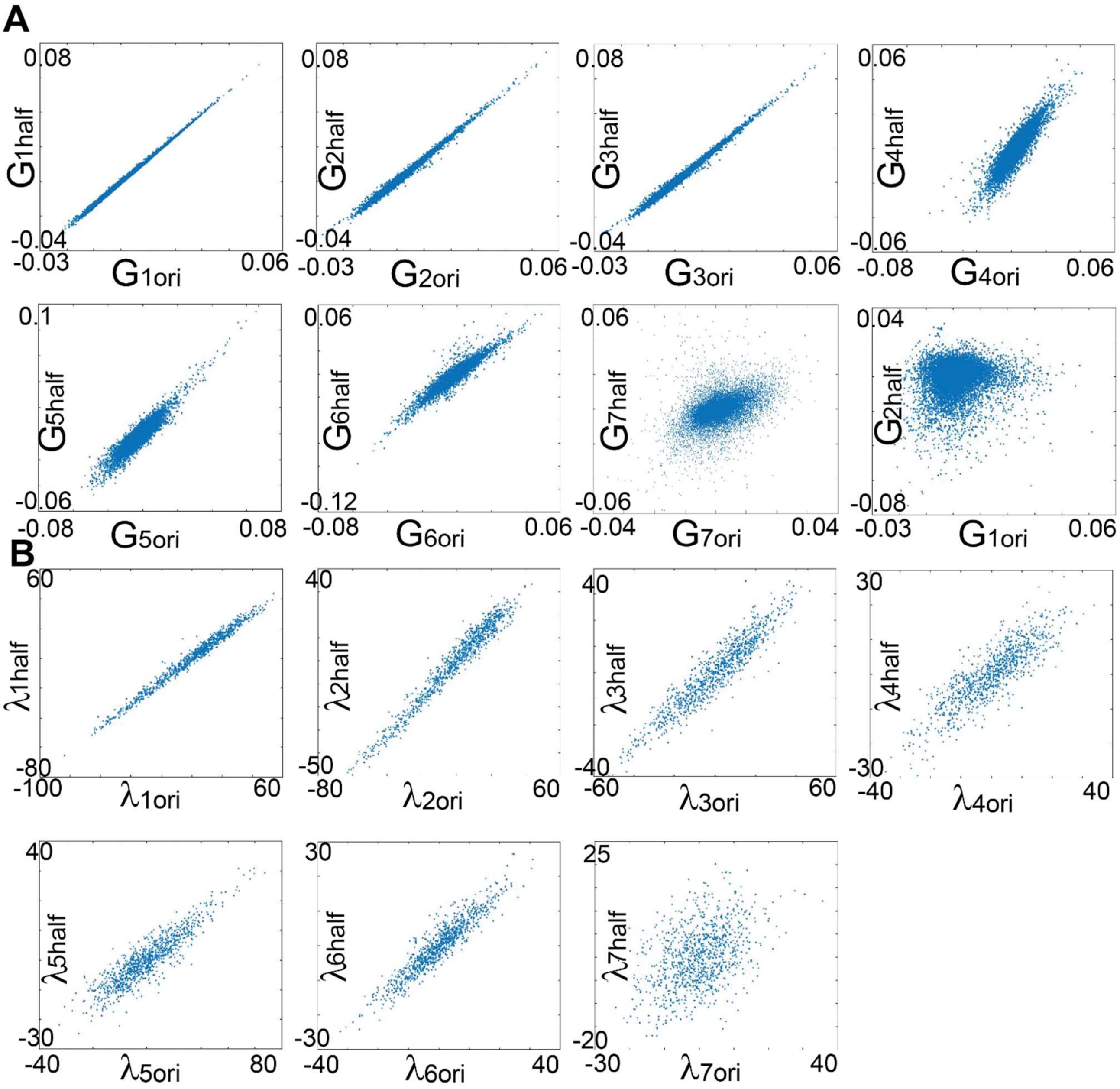

Fig. S6 Unbalanced processes remained the same when either the entire dataset or a subset of the dataset comprising of only half genes in the dataset were used for the analysis. The weights of the transcripts (Giα; **A**) and the amplitudes of the unbalanced processes (λα; **B**) were found to be similar when either the entire dataset (**ori** dataset, 951 samples, and 44827 genes) or only half the subgroup of genes from the dataset (**half** dataset, 951 samples, 22351 genes) were analyzed, as indicated by high correlation of the scatter plots. As a negative control, we show an example of poor correlation when we compare the weights of two processes 1 and 2 from the two datasets (G2 vs G1, (**A**), **lower panel, right plot).**

Figure S7

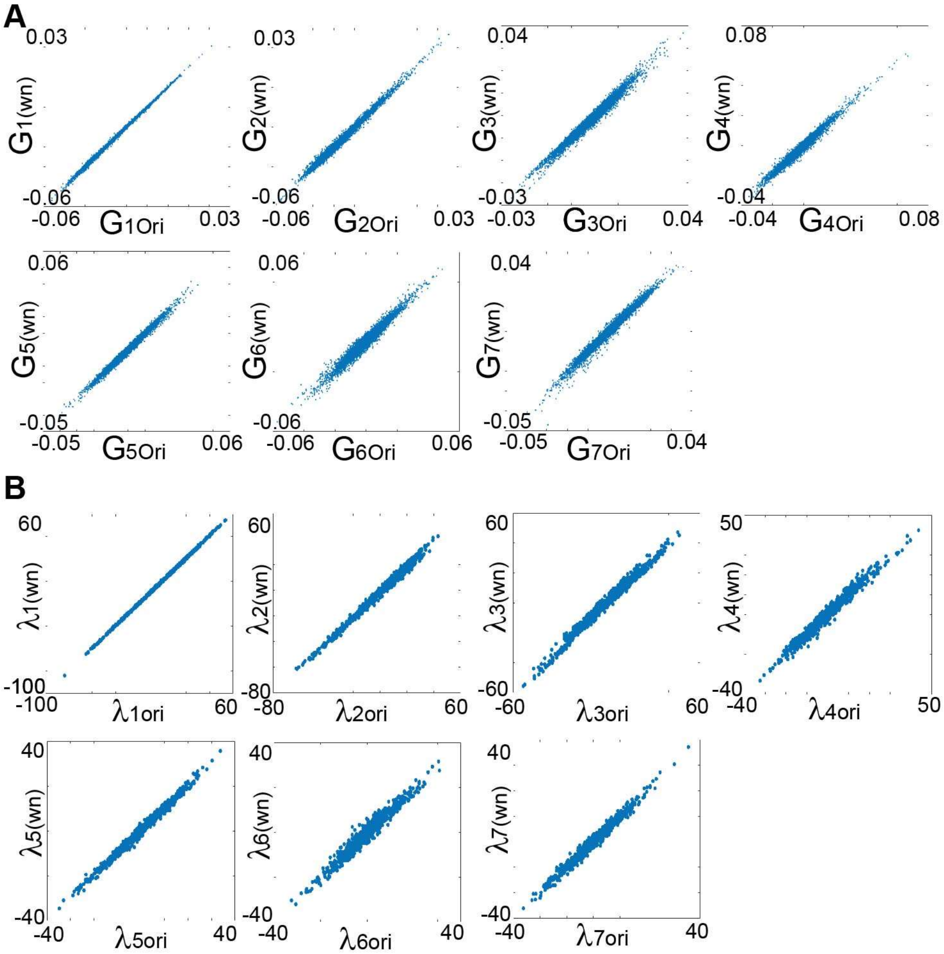

Fig. S7 Unbalanced processes remained the same when either the entire dataset or the dataset without normal samples was used for the analysis. The weights of the transcripts (Gi, (**A**)) and the amplitudes of the unbalanced processes (**B**) were found to be similar when either the entire dataset (**ori** dataset, 951 samples, and 44827 genes) or the dataset without normal samples (**wn** dataset, 737 samples, 44827 genes) were analysed, as indicated by the high correlation of the scatter plots. The correlation coefficient, R, was found to be greater than 0.9 when the weights of the transcripts and amplitudes were compared.

Figure S8

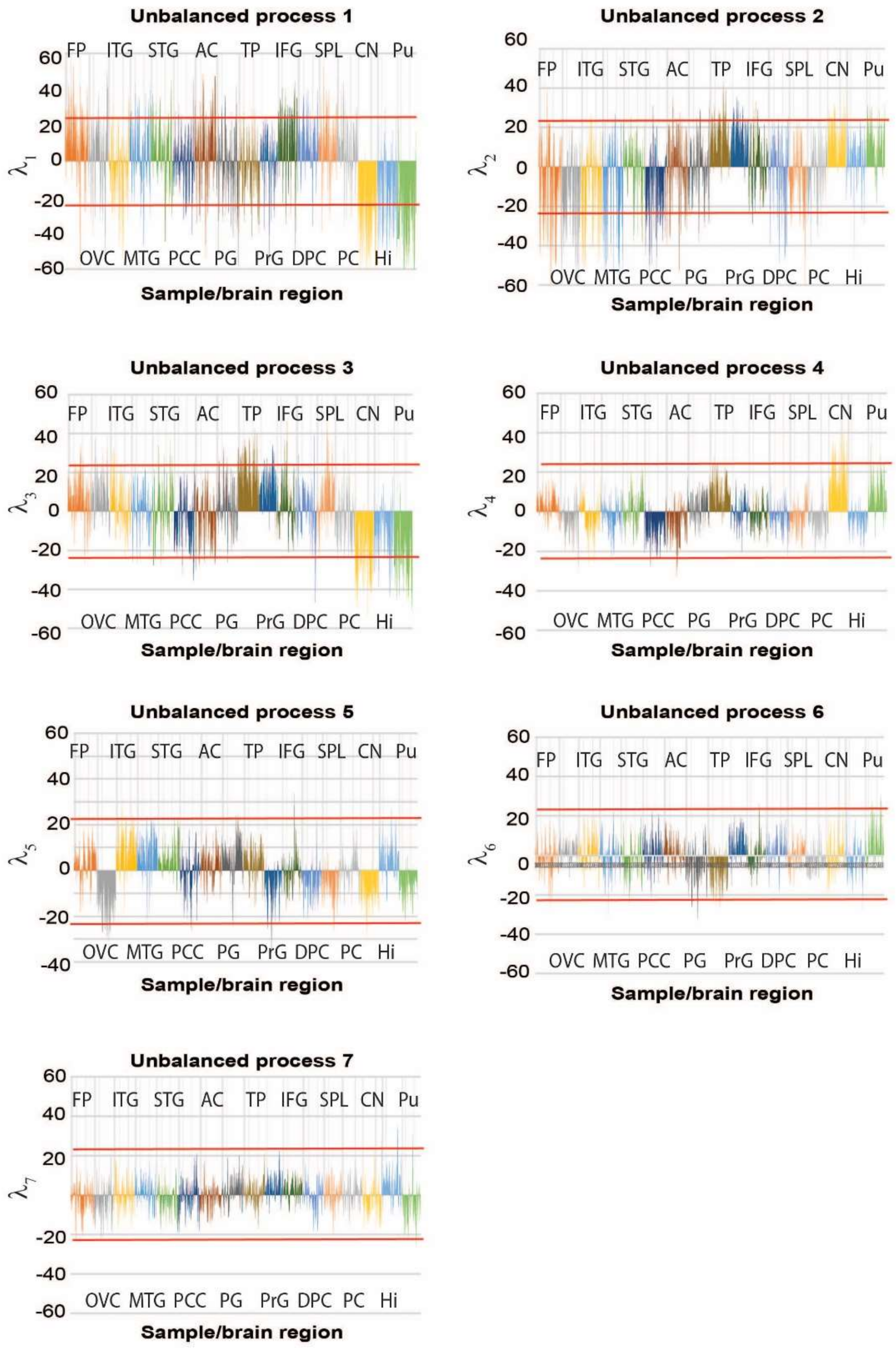

Fig. S8 Amplitudes of the unbalanced processes in the 951 brain samples. The figure presents amplitudes, $\lambda_\alpha(k)$, of the 7 unbalanced processes for all 951 brain samples. Brain regions included: frontal pole (FP), occipital visual cortex (OVC), inferior temporal gyrus (ITG), middle temporal gyrus (MTG), superior temporal gyrus (STG), posterior cingulate cortex (PCC), anterior cingulate (AC), parahippocampal gyrus (PG), temporal pole (TP), precentral gyrus (PrG), inferior frontal gyrus (IFG), dorsolateral prefrontal cortex (DPC), superior parietal lobule (SPL), prefrontal cortex (PC), caudate nucleus (CN), hippocampus (Hi) and putamen (Pu). The red lines mark threshold limits.

Figure S9

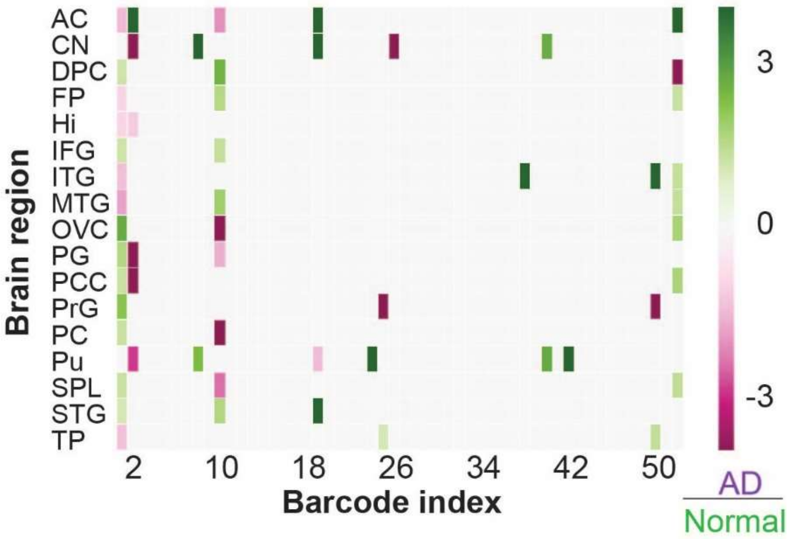

Fig. S9 Significance of each barcode in each brain region. Pink color indicates higher significance of the barcode in the diseased samples, while green color indicates higher significance in the normal samples. The figure demonstrates that in the same brain region, some of the barcodes are enriched in the normal samples, whereas others are enriched in the diseased samples. For example, in CN, barcode 8 is enriched in the normal samples, whereas barcode 2 is enriched in the diseased samples.
